# Integrated Photonic Biosensors: Enabling Next-Generation Lab-on-a-Chip Platforms

**DOI:** 10.3390/nano15100731

**Published:** 2025-05-13

**Authors:** Muhammad A. Butt, B. Imran Akca, Xavier Mateos

**Affiliations:** 1 Institute of Microelectronics and Optoelectronics, Warsaw University of Technology, Koszykowa 75, 00-662 Warsaw, Poland; 2 LaserLab, Department of Physics and Astronomy, VU University, De Boelelaan 1081, 1081 HV Amsterdam, The Netherlands; 3 Fisica i Cristal⋅lografia de Materials (FiCMA), Universitat Rovira i Virgili (URV), Marcel⋅li, Domingo 1, 43007 Tarragona, Spain

**Keywords:** photonic sensors, lab-on-chip, integrated optics, bio-diagnostics

## Abstract

Integrated photonic biosensors are revolutionizing lab-on-a-chip technologies by providing highly sensitive, miniaturized, and label-free detection solutions for a wide range of biological and chemical targets. This review explores the foundational principles behind their operation, including the use of resonant photonic structures such as microring and whispering gallery mode resonators, as well as interferometric and photonic crystal-based designs. Special focus is given to the design strategies that optimize light–matter interaction, enhance sensitivity, and enable multiplexed detection. We detail state-of-the-art fabrication approaches compatible with complementary metal-oxide-semiconductor processes, including the use of silicon, silicon nitride, and hybrid material platforms, which facilitate scalable production and seamless integration with microfluidic systems. Recent advancements are highlighted, including the implementation of optofluidic photonic crystal cavities, cascaded microring arrays with subwavelength gratings, and on-chip detector arrays capable of parallel biosensing. These innovations have achieved exceptional performance, with detection limits reaching the parts-per-billion level and real-time operation across various applications such as clinical diagnostics, environmental surveillance, and food quality assessment. Although challenges persist in handling complex biological samples and achieving consistent large-scale fabrication, the emergence of novel materials, advanced nanofabrication methods, and artificial intelligence-driven data analysis is accelerating the development of next-generation photonic biosensing platforms. These technologies are poised to deliver powerful, accessible, and cost-effective diagnostic tools for practical deployment across diverse settings.

## 1. Introduction

In recent years, the intersection of photonic biosensing and lab-on-a-chip (LOC) technologies has emerged as one of the most exciting frontiers in analytical science [1,2]. Together, these fields are redefining how we approach diagnostics by offering tools that are not only highly sensitive and rapid, but also compact and portable. At the heart of this progress are integrated photonic biosensors, which exploit the unique properties of light to detect biological analytes with exceptional specificity, often without the need for labels or amplification steps [3]. Photonic biosensors work by guiding light through micro- and nanoscale structures, such as waveguides [4], resonators [5], and interferometers [6], and monitoring subtle changes in the light’s behavior as it interacts with a target analyte [7,8,9]. Because these interactions often result in shifts in refractive index [10], phase [11], or intensity [12], they can be precisely measured to detect even trace levels of biomolecules [13,14]. When these optical components are combined with microfluidics [15], the result is a LOC platform, which is a miniaturized, self-contained system capable of performing complex biochemical analyses on a single chip [16].

This integration brings several powerful advantages. LOC systems allow for precise fluid handling, minimal reagent consumption, and faster reaction times, all while reducing the size and cost of diagnostic devices. Photonic biosensors, on the other hand, offer fast, real-time readout, high multiplexing potential, and immunity to electromagnetic interference [17,18]. Together, these technologies are making it possible to move diagnostics out of centralized labs and into clinics, homes, and field settings and thereby drive the global shift toward point-of-care and personalized healthcare [19]. This review builds upon these foundations by highlighting the emerging innovations that define the next generation of integrated photonic biosensors. These include advanced complementary metal-oxide-semiconductor (CMOS)-compatible fabrication techniques, hybrid platforms incorporating silicon nitride and organic lasers, optofluidic photonic crystal cavities, integrated detector arrays, and machine learning-enabled signal processing. These developments go beyond incremental improvements by enabling real-time, low-power, and multiplexed detection capabilities within highly miniaturized systems. As such, they are paving the way for biosensing platforms that are not only more sensitive and robust but also accessible, scalable, and tailored for deployment in real-world scenarios including remote and resource-limited environments.

A major factor driving the adoption of integrated photonics is the growing maturity of CMOS-compatible fabrication technologies, particularly in silicon photonics [20]. This allows for large-scale production and co-integration of optical sensing elements with electronic circuits for signal processing and communication. Other material platforms, such as silicon nitride, indium phosphide, and polymers, are also being explored to expand operational wavelengths, reduce optical losses, and introduce new functionalities [21,22,23]. The field is evolving rapidly, with research pushing the limits of sensitivity, selectivity, and scalability [24,25]. There is growing interest in hybrid platforms [26] that combine photonics with plasmonics [27,28], microelectromechanical systems (MEMSs) [29], or artificial intelligence (AI) [30,31] to enhance performance and automation. These advancements are unlocking new applications in clinical diagnostics, environmental monitoring, food safety, and beyond [32,33].

Recent developments in integrated photonic biosensors are increasingly characterized by their enhanced functionality, scalability, and real-world applicability of next-generation platforms [9,34,35,36]. For example, cascaded microring resonators fabricated with subwavelength grating waveguides have achieved unprecedented sensitivity and low detection limits while remaining compatible with CMOS processes [37,38,39]. Similarly, optofluidic photonic crystal cavities have been shown to significantly boost light–matter interaction, enabling detection of biomolecules at extremely low concentrations [40,41]. The integration of these photonic devices with microfluidic platforms, real-time signal processing modules, and artificial intelligence for data interpretation marks a shift from proof-of-concept demonstrations to practical, scalable diagnostic solutions. Furthermore, the incorporation of photonic biosensors into wearable systems and organ-on-chip models reflects the growing emphasis on personalized, continuous, and decentralized healthcare [42,43,44]. These advancements underscore the emerging direction of the field and provide strong motivation for a comprehensive review focused on the technological foundations and recent innovations defining this next generation of lab-on-a-chip biosensing platforms.

In this review, we present an in-depth examination of the evolving landscape of integrated photonic biosensors as key enablers of next-generation LOC systems, beginning with a detailed analysis of core optical sensing principles such as resonant structures and interferometric techniques (Section 2). We then explore the materials, fabrication strategies, and integration methods that underpin these devices (Section 3). Building on this foundation, we highlight recent technological advances (Section 4), including silicon photonics, optofluidic photonic crystal cavities, CMOS-compatible detector arrays, and other innovations that are shaping the future of LOC platforms. Diverse real-world applications in medical diagnostics, environmental monitoring, and food quality assurance are also discussed (Section 5), and conclude by discussing the current challenges and emerging opportunities that will define the next generation of integrated biosensing technologies.

## 2. Principles of Photonic Biosensing

Photonic biosensors are advanced analytical tools that utilize the interaction between light and biological substances to detect and quantify analytes with exceptional sensitivity [34]. These devices work by monitoring changes in the optical properties of a system, such as refractive index shifts, variations in light absorption, or changes in fluorescence, resulting from biological interactions. When a target analyte binds to a functionalized sensor surface, it alters the local optical environment. This change is then translated into an optical signal, which enables precise and often real-time detection. Due to their ability to operate without labels and their compatibility with miniaturization, photonic biosensors are increasingly used in fields such as clinical diagnostics, environmental monitoring, and food safety analysis.

Evanescent field sensing is a core principle in photonic biosensing technologies [45]. This technique relies on the evanescent wave—an electromagnetic field that extends slightly beyond the surface of an optical waveguide or fiber. As light propagates through a waveguide, a fraction of its energy extends into the surrounding environment, allowing interaction with nearby materials. Because the evanescent field is highly sensitive to variations in the local refractive index [46,47], it enables detection of molecular events occurring at or near the waveguide surface. For instance, when biological molecules bind to receptors immobilized on the sensor’s surface, they alter the refractive index in the immediate vicinity [48]. This interaction results in detectable changes in the light’s properties, such as its phase, intensity, or wavelength. Well-established technologies like surface plasmon resonance (SPR) [49] and optical waveguide-based sensors operate on this principle. The method enables real-time, label-free detection and is particularly well-suited for use in integrated, miniaturized lab-on-a-chip platforms [45].

**Figure 1:**
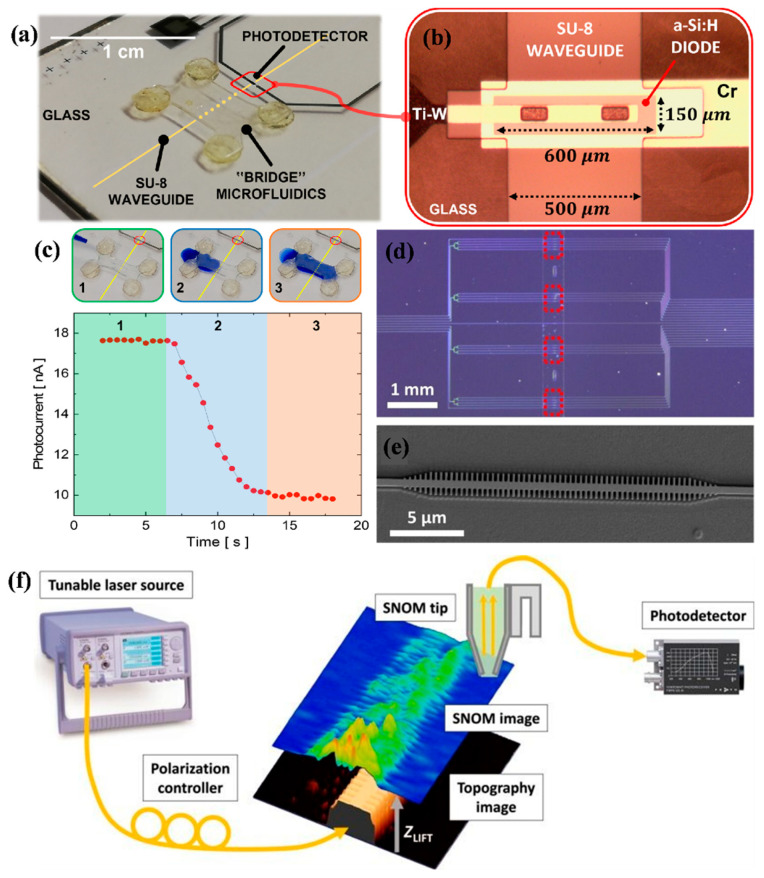
(**a**) Image of the complete fabricated system [50]. (**b**) Magnified top view of the integrated detector, highlighting structural details [50]. (**c**) Three distinct filling phases using blue-dyed deionized water for visualization, while the graph below displays the corresponding time-dependent response of the sensor’s photocurrent as the bridge channel is filled with a milk sample [50]. (**d**) Optical microscope view of the complete photonic chip, where red dotted squares denote the positions of the individual PBG sensor arrays [51]. (**e**) Enlarged SEM image illustrating the structural details of a single PBG sensing element located within the chip [51]. (**f**) A schematic illustrates the SNOM setup and the corresponding measurement process [51].

Buzzin et al. introduced a novel, integrated optical biosensing platform that combined both the light–sample interaction and detection functions within a single, compact glass chip [50]. Unlike conventional systems, where the light source, interaction zone, and detector are assembled as separate units, this monolithic design offered a more streamlined, low-cost, and user-friendly alternative. The core sensing mechanism relied on the interaction of evanescent waves with the complex refractive index of a liquid mixture. Since this index varies based on the mixture’s physical and chemical characteristics, it enables sensitive detection of compositional changes. Utilizing thin-film microelectronic techniques, the prototype was constructed through a sequence of four lithographic steps tailored to the proposed design. The complete system was integrated within a compact footprint. An overview of the assembled device is provided in Figure 1a, while Figure 1b offers a close-up microscopic view of the detection area, emphasizing the detailed geometry of the sensing region.

To evaluate the sensor’s dynamic response, a preliminary test was conducted by continuously monitoring the waveguide–sample interaction during the filling of the microfluidic channel with milk. The evolution of the photocurrent signal, shown in Figure 1c, captures this process, with illustrative images of the filling sequence (using blue-dyed deionized water as a demonstrative fluid) presented at the top of the figure. The test revealed three distinct phases: initially, the channel remained empty, and the system maintained a stable baseline photocurrent (phase 1). As the liquid began to enter the channel and partially overlapped the underlying SU-8 waveguide (phase 2), interaction with the evanescent field caused a measurable drop in the photocurrent. Once the channel was filled and the fluid stabilized (phase 3), the signal reached a new steady-state level, lower than the initial baseline. The prototype achieved a sensitivity of approximately 139 fA per (g/dL) and a detection limit as low as 14 ppm, surpassing the performance of current commercial devices [50].

To enable label-free protein detection, a photonic bandgap (PBG) biosensor was developed and tested (Figure 1d) [51]. The SEM image of one of the chips is shown in Figure 1e). The sensing mechanism relied on the interaction between target analytes and the evanescent field that extends from the PBG structure into the cladding layer. A customized scanning near-field optical microscopy (SNOM) setup was employed to characterize the PBG structures (Figure 1f). In this configuration, light from a tunable laser source was coupled into the photonic chip using a cleaved single-mode optical fiber. The near-field optical signal generated by the PBG structure was then collected using a bent optical fiber tip, which was pre-mounted on a tuning fork operating in tapping mode. This arrangement enabled high-resolution mapping of the evanescent field distribution near the sensor surface. This finding emphasized the importance of placing biorecognition elements as close as possible to the PBG surface to achieve maximum sensitivity. To optimize this interaction, the biosensor surface was biofunctionalized using half-antibodies specific to bovine serum albumin (BSA), immobilized through a UV-assisted method. The use of half-antibodies effectively reduced the thickness of the recognition layer to around 2.5 nm, promoting a stronger overlap with the evanescent field and ensuring favourable orientation of the binding sites toward the analyte. Following functionalization, the PBG biosensor successfully performed real-time, direct detection of the BSA antigen.

Apart from biosensing, evanescent field absorption sensing on a chip is an advanced technique for gas detection that leverages the interaction between the evanescent field and gas molecules [52]. In this approach, light is guided through a waveguide or optical fiber, and part of the light energy extends into the surrounding medium as an evanescent field. When gas molecules are present near the sensor surface, they absorb specific wavelengths of light from the evanescent field [53]. The extent of this absorption depends on the concentration of the target gas and its absorption spectrum, allowing for precise detection. By measuring changes in the intensity or wavelength of the transmitted light, the concentration of the gas can be determined in real-time [54]. The sensitivity of evanescent field absorption sensors is enhanced by functionalizing the waveguide surface to selectively interact with the target gas molecules, allowing for high selectivity and fast detection [55]. These sensors are particularly valuable for compact, on-chip gas detection systems due to their small size, label-free detection capability, and ability to detect gases at trace levels with high sensitivity [56]. This section explores two of the most widely adopted sensing mechanisms in integrated photonic biosensors—resonant photonic structures and interferometric techniques.

### 2.1. Resonant Structures

**Figure 2:**
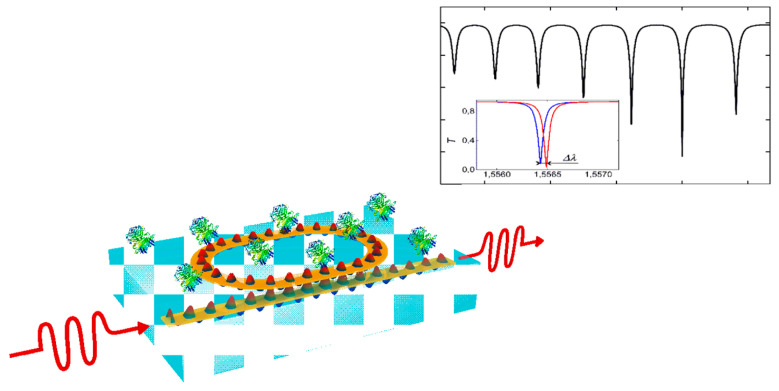
A schematic of a racetrack ring resonator used for biosensing is presented, along with its output spectrum, which demonstrates a resonance wavelength shift in response to changes in the surrounding medium’s refractive index [63].

Resonant photonic structures form another powerful class of biosensing mechanisms. These structures include microring resonators (MRR) [57,58,59], whispering gallery mode (WGM) resonators [60,61], and photonic crystal cavities [16,62]. They operate on the principle that certain optical structures can confine light and cause it to resonate at specific wavelengths or frequencies. The resonant frequency is highly sensitive to changes in the surrounding refractive index. When a biological analyte binds to the surface of such a resonator, it alters the local optical environment, causing a detectable shift in the resonant frequency (Figure 2) [63]. These shifts are typically very narrow and can be measured with high accuracy, allowing for the detection of even minute concentrations of analytes [64]. For example, photonic crystal biosensors leverage periodic nanostructures to create photonic band gaps, enabling strong confinement and enhanced sensitivity to refractive index changes [65]. The high Q-factors of these resonators result in superior performance in terms of sensitivity and specificity.

Beyond the basic principle of resonance, the effectiveness of a photonic resonator as a biosensor is determined by how tightly it confines light and how strongly that light interacts with its surroundings [48]. In these devices, light recirculates many times around a closed path or within a defect region, creating an intense standing wave whose evanescent tail extends into the surrounding medium. Any change in the local refractive index, such as when biomolecules bind to the resonator surface, modifies the optical path length and shifts the resonant condition [59,66]. This interaction can be controlled by choosing the resonator geometry (ring, disk, or photonic-crystal defect), adjusting the coupling strength to the bus waveguide (to achieve critical coupling), and selecting materials with low intrinsic loss [16,58]. The trade-off between spectral resolution (narrow linewidth) and device footprint or bandwidth is captured by the resonator’s quality factor and free-spectral range. By optimizing these parameters in concert with surface functionalization strategies, resonant photonic structures can be engineered for highly sensitive, label-free detection across a range of biochemical targets [6].

Yoo et al. introduced a compact, near-infrared optical biosensing platform fabricated on a silicon-on-insulator (SOI) wafer [59]. This LOC system integrates an MRR with an on-chip spectrometer, effectively eliminating the requirement for external optical spectrum analyzers. The biosensor features a symmetric add-drop MRR design with a free spectral range (FSR) close to 19 nm and demonstrates a bulk sensitivity of approximately 73 nm/RIU. Spectral information from the drop port is retrieved using a spatial-heterodyne Fourier transform spectrometer (SHFTS), offering a resolution of about 3.1 nm across a 50 nm bandwidth. The overall system achieved a detection limit of 0.042 RIU [59].

**Figure 3:**
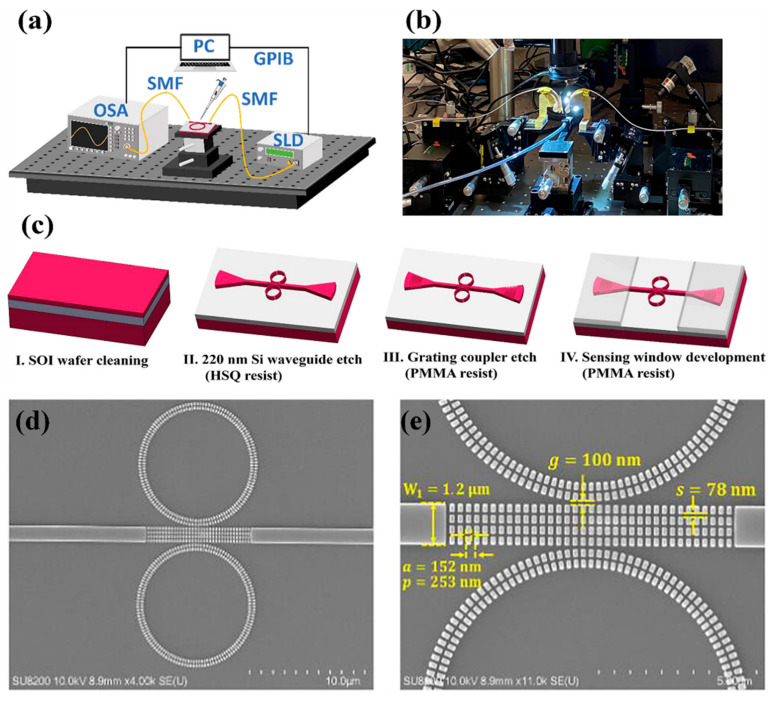
(**a**) Illustration of the experimental setup layout. (**b**) Image of the measurement platform. (**c**) Step-by-step overview of the device fabrication process. (**d**) SEM image showing the SWG-CMRR device. (**e**) Close-up of the SWG structure with corresponding fabrication parameters [37].

To further enhance performance, silicon-on-insulator subwavelength grating (SWG) waveguides were combined with cascaded MRRs, significantly boosting light–matter interaction. This integration led to marked improvements in sensitivity, detection limits, and spectral coverage compared to conventional strip or slot waveguide MRRs. In a separate effort, Cheng et al. designed and experimentally validated a novel biochemical sensor based on this architecture [37]. Their device, featuring cascaded MRRs integrated with SWG waveguides (SWG-CMRRs), was tested using a silicon photonic setup employing vertical coupling and LABVIEW-based signal processing tailored for biochemical sensing. Figure 3a,b show the experimental arrangement for characterizing the SWG-CMRR sensors, while Figure 3c outlines the fabrication process. Figure 3d presents a top-view scanning electron microscope (SEM) image of the completed sensor, and Figure 3e provides a close-up SEM of the SWG multibox structure with corresponding fabrication measurements.

This sensor achieved an impressive refractive index sensitivity of 810 nm/RIU and a detection limit of 2.04 × 10^−5^ RIU. For sodium chloride solutions, the measured concentration sensitivity is 1430 pm/% with a detection limit of 0.04%. The sensor also exhibited an FSR of 35.8 nm and a quality factor (Q) of 1.9 × 10^3^. By combining the advantages of cascaded MRRs with subwavelength grating waveguides, the sensor demonstrated unprecedented sensitivity, making it an excellent candidate for advancing biochemical sensing applications, healthcare diagnostics, and environmental monitoring systems [37].

WGM resonators have gained significant attention for biosensing applications due to their exceptional sensitivity, enabling label-free detection. Despite considerable progress in academic research, their practical impact has remained limited. Kim et al. presented a novel platform for on-chip WGM sensors integrated with microfluidic channels, designed to overcome these limitations [67]. The key innovation lies in incorporating silicon nanoclusters into micro-resonators, which act as stable active compounds. This integration allowed the sensor chip to be operated remotely, simplifying both its integration and connection to external systems. Additionally, silicon nanoclusters, with their broad absorption cross-section over a wide wavelength range, enabled active sensing using a top-illumination scheme with an LED pump. This approach significantly reduced the complexity and cost of the measurement setup. The sensor also featured a nano-slot structure with a 25 nm gap width, embedded within the resonator. This design selectively detected target biomolecules with enhanced sensitivity due to the strongly confined mode field.

**Figure 4:**
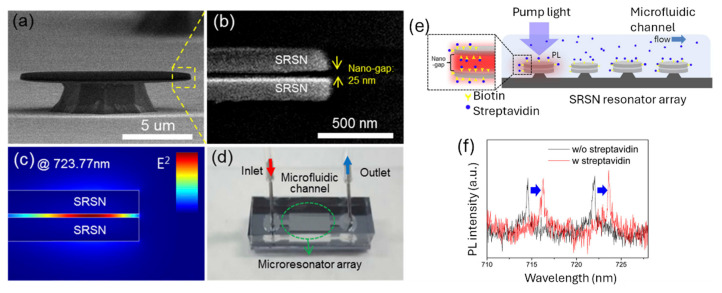
(**a**) SEM image of a disk resonator. (**b**) Zoomed-in view of the yellow box in (**a**), highlighting two separate disks and a 25 nm gap. (**c**) Transverse magnetic (TM) fundamental mode intensity profile, simulated in an aqueous environment using COMSOL Multi-Physics. (**d**) Photograph of the on-chip sensor, featuring disk resonators integrated into a microfluidic channel. (**e**) Schematic of the microfluidic channel for detecting streptavidin binding to biotin-immobilized SRSN resonators. (**f**) PL spectrum of biotinylated SRSN resonator before (black line) and after (red line) streptavidin interaction in an aqueous environment [67].

Figure 4a shows the SEM image of the fabricated SRSN disk resonators, which have a 12 µm diameter and a 120 nm thickness. The magnified image in Figure 4b highlights the 25 nm gap. Numerical simulations using COMSOL Multi-Physics reveal that, in a water environment, 8.6% of the total energy of the fundamental mode is confined within this gap (see inset in Figure 4c). This confinement leads to a 6.5-fold enhancement in sensitivity, specifically for events occurring within the gap. As shown in Figure 4d, the fabricated resonator arrays are integrated into a microfluidic channel, creating a compact optical sensor.

The sensor’s sensitivity is confirmed by measuring the peak shift in the streptavidin-biotin complex, as shown in Figure 4e. Before starting the experiment, the SRSN resonators were pre-treated with biotin, as described in the methods section. As streptavidin flows through the microfluidic channel in DPBS, it binds to the biotin on the resonator surface, causing a shift in the resonance frequency. The photoluminescence (PL) spectra, recorded before and after introducing 144 nM streptavidin, are shown as black and red lines in Figure 4f, respectively. The red spectrum, obtained after the binding has reached saturation, shows a peak shift of 1.7 nm, which corresponds to a sensitivity of 0.012 nm/nM for the sensor. This novel platform represented a significant step forward in making WGM-based biosensors more practical and cost-effective for real-world applications [67].

### 2.2. Interferometric Techniques

Interferometry is a well-established and highly sensitive optical technique used in many types of photonic biosensors [6,68]. Interferometric biosensors work by splitting a coherent light beam into two separate paths: a sensing arm and a reference arm. The sensing arm interacts with the biological sample, while the reference arm remains unaffected. Upon recombination, the light from both arms produces an interference pattern [35]. Any biological interaction, such as analyte binding, changes the refractive index in the sensing arm, altering the optical path length and resulting in a phase shift. This phase shift modifies the interference pattern and serves as a quantitative indicator of the analyte’s presence and concentration. Common interferometric devices include Mach–Zehnder’s interferometers (MZI) [69,70,71,72], Young’s interferometers [73,74,75], and Michelson’s interferometers [76,77]. These sensors offer real-time, label-free detection with extremely high sensitivity, making them ideal for applications requiring precise measurement of molecular interactions, including kinetics and affinity studies.

In practice, interferometric biosensors leverage a variety of design and readout strategies to maximize sensitivity and robustness [11,78,79]. Integrated photonic Mach–Zehnder devices often incorporate balanced detection or phase modulation schemes to suppress common-mode noise and drift, while multi-arm interferometers and arrayed waveguide gratings enable simultaneous monitoring of multiple analytes [79]. Coherent detection techniques, such as heterodyne or phase-stepped interrogation, extend the dynamic range and improve resolution down to milliradian-level phase shifts [80]. Temperature and polarization control are also critical design considerations, frequently addressed through on-chip reference channels or athermal waveguide designs [81]. Finally, the planar geometry of interferometers lends itself readily to microfluidic integration, allowing precise fluid handling and multiplexed sampling in a compact footprint [79,82]. These enhancements collectively make interferometric platforms not only highly sensitive but also versatile and scalable for real-world biosensing applications.

**Figure 5:**
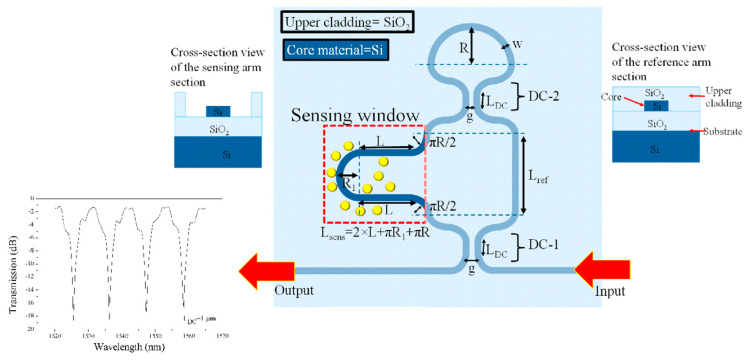
The 2D schematic of the a-LT-MZI structure. Inset: cross-sections of the reference (right) and sensing (left) arms, with yellow markers indicating analytes. The output port displays the interference pattern as a function of wavelength [17].

For instance, M.A. Butt presented a compact and sensitive refractive index sensor based on an asymmetric loop-terminated Mach–Zehnder interferometer (a-LT-MZI) on a SOI platform (Figure 5) [17]. By integrating a Sagnac loop, the device reduced the effective optical path length by half, supporting miniaturization for integrated photonic systems. The interference pattern as a function of wavelength is also shown in Figure 5. With a pathlength difference (ΔL) of 24.35 µm, the sensor achieved a sensitivity of 261 nm/RIU. Incorporating a subwavelength grating (SWG) waveguide in the sensing arm enhances light–matter interaction, boosting sensitivity to 510 nm/RIU. This design offers an efficient, high-performance solution for compact optical biosensors.

Furthermore, a high-sensitivity, label-free optical biosensor based on a MZI was introduced, featuring a highly dispersive one-dimensional (1D) photonic crystal integrated into one of its arms [83]. The incorporation of a slow light photonic crystal significantly enhanced the device’s performance, with sensitivity directly scaling with the length of the slow light region. Numerical analysis revealed that a 16 μm-long slow light section can achieve a sensitivity of 115,000 rad/RIU-cm—over seven times greater than that of conventional MZI biosensors with millimeter-scale sensing arms. Experimentally, the sensor demonstrated a bulk refractive index sensitivity of 84,000 rad/RIU-cm. In addition to refractive index measurements, the platform has also been successfully applied for nucleic acid detection [83].

**Figure 6:**
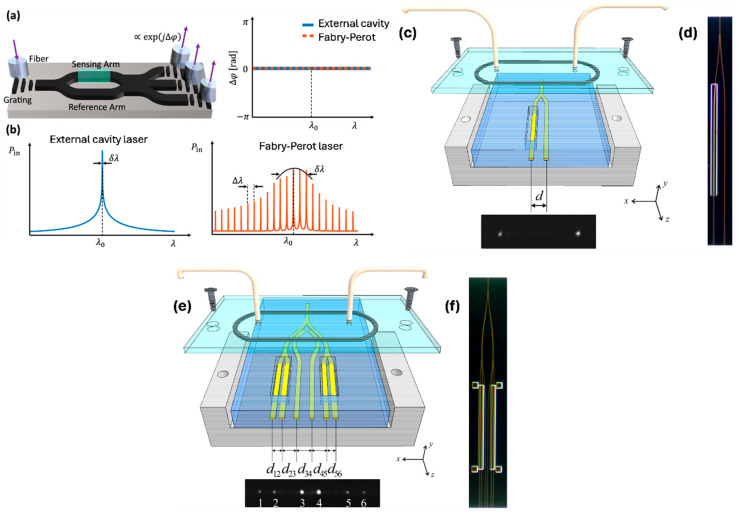
(**a**) Schematic of a coherently detected balanced MZI, where phase difference between the reference and sensing arms is extracted from three outputs. The wavelength response is flat when arm lengths are balanced [68]. (**b**) Approximate spectra of a narrow-linewidth external cavity laser and a Fabry–Perot laser, showing center wavelengths, linewidths, and spectral spacings [68]. (**c**) Illustration of a Y-junction Young’s interferometer and its collimated output in the far-field region [73]. (**d**) Close-up view of the Y-junction structure captured under a microscope [73]. (**e**) Diagram of a corporate-feed Young’s interferometer showing its collimated outputs in the far-field region [73]. (**f**) Microscopic image of the corporate-feed multichannel structure [73].

Among the various designs, interferometric sensors stand out for their simplicity and compatibility with fixed-wavelength laser readouts, alongside strong detection capabilities. However, a common drawback of these systems is a drop in sensitivity when not operated at their optimal phase point. To overcome this, coherent detection methods have emerged as a compelling solution. Leuermann et al. reported the first demonstration of sub-nanogram per milliliter detection limits for C-reactive protein (CRP) immunoassays using a coherent detection approach [68]. Furthermore, by carefully matching the optical path lengths in the sensor arms, stable operation using low-cost Fabry–Perot laser sources was enabled at telecommunications wavelengths. Figure 6a illustrates the operating principle of MZI sensors, where incident light is split into two single-mode waveguides—designated as the sensing and reference arms. The reference waveguide is encapsulated in silicon dioxide, providing a stable environment in which the optical mode accumulates a constant phase shift. In contrast, the sensing arm is exposed to the analyte, allowing interaction between the guided light and the external medium, which induces an analyte-dependent phase change. In conventional MZI designs, the two optical paths are recombined using structures such as Y-junctions or multi-mode interference (MMI) couplers. The interference pattern at the output is determined by the phase difference between the two arms. However, this configuration is inherently sensitive to relative intensity noise and often suffers from phase ambiguity and diminished sensitivity, particularly when the system is not biased at its quadrature point. To overcome these drawbacks, a coherently read MZI architecture has been introduced. In this approach, the sensing and reference signals were recombined using a balanced directional coupler, as shown in Figure 6a, enabling more robust and noise-resilient detection.

To investigate the influence of laser linewidth on the sensor’s performance, experiments were conducted using two types of laser sources—one high-end and one low-cost. The high-quality reference source was the WSL-100 external cavity laser (ECL) from SANTEC, Japan, known for its narrow linewidth of 100 kHz. This single-mode laser, priced at approximately EUR 10,000, served as the performance benchmark (Figure 6b). For comparison, a handheld Fabry–Perot laser (FPL) was selected as a budget-friendly alternative. This device, purchased on Amazon for roughly EUR 34, operated at a center wavelength of 1.543 µm, with a mode spacing of 1.36 nm and a broad spectral bandwidth of 450 GHz (Figure 6b). These two sources enabled a direct assessment of how spectral purity impacts interferometric biosensor operation. Detection limits below 300 pg/mL for C-reactive protein (CRP) were achieved using a high-performance reference laser source. Even with a cost-effective Fabry–Perot laser, a limit of detection (LOD) below 2 ng/mL was attained. These advancements are considered a significant step toward the development of compact, low-cost, point-of-care (POC) biosensing technologies [68].

Two integrated Young’s interferometer (YI) sensors utilizing long-range surface plasmon polariton (LRSPP) waveguides were introduced by Wong et al. [73]. The first design employed a Y-junction splitter for single-channel operation, whereas the second featured a corporate feed structure enabling multi-channel functionality. This multi-channel configuration facilitated real-time, independent monitoring of refractive index changes across several channels based on phase variation. In both configurations, interference patterns were produced in the far field as the output beams from the waveguides diverged and overlapped. These patterns were analyzed using the fast Fourier transform (FFT) to extract phase-related data. To evaluate sensing performance, a sequence of solutions with progressively higher refractive indices was introduced into the sensing channels [73].

As shown in Figure 6c,d, the Y-junction waveguide functions as a single-channel Young’s interferometer. To define a fluidic channel, the top cladding was etched over one arm of the Y-junction, exposing the Au stripe surface. The other arm remained cladded, acting as a reference channel. When a refractive index change occurred in the sensing channel—whether from alterations in the bulk refractive index of the fluid or from the formation of a biochemical layer on the waveguide—a spatial shift (Δx) appeared in the interference pattern captured on the camera surface. Figure 6e,f depict the corporate-feed multichannel configuration, consisting of four sensing channels, which were defined by etches on waveguides 1, 2, 5, and 6. Two reference channels are provided by the cladded waveguides 3 and 4. The corporate feed structure was constructed by cascading two Y-junction splitters to form a primary Y-junction. To facilitate referencing, a pair of couplers is integrated into each branch of the initial Y-junction to extract a small portion of the light. LRSPP-based YIs achieved a detection limit of approximately 1 × 10^−6^ RIU, marking a significant improvement over a similar attenuation-based sensing approach [73].

## 3. Integration with Lab-on-Chip Systems

The incorporation of photonic biosensors into LOC platforms represents a transformative advancement in analytical technology, achieved by integrating optical components directly with microfluidic networks [84]. This fusion yields compact, efficient, and highly functional diagnostic systems tailored for modern applications [1,85,86]. One of the most impactful outcomes of this integration is miniaturization, which enables the development of portable devices suitable for on-site or point-of-care testing [87]. Additionally, the design significantly reduces the volume of samples and reagents required, making it particularly advantageous for analyses involving rare, costly, or limited biological materials. Another key strength is the ability to perform multiplexed detection, allowing simultaneous identification of multiple analytes in a single assay, thereby increasing throughput and diagnostic precision [88,89]. Equally important is the speed of analysis, as optical sensing methods offer rapid detection, a crucial factor in urgent scenarios such as infectious disease outbreaks. By merging photonic and microfluidic technologies, LOC systems are not only becoming more accessible and user-friendly but also more powerful in addressing real-time diagnostic and monitoring challenges across a range of fields [90].

Table 1 outlines the fundamental components of LOC systems that incorporate photonic biosensors, providing a comprehensive overview of each element’s role, function, and common materials. These components work synergistically to enable miniaturized, efficient, and high-performance analytical devices suitable for a wide range of biomedical and environmental applications. From microfluidic networks that precisely handle fluid samples to biorecognition elements that confer molecular specificity, each part plays a critical role in the operation and reliability of the overall system. Additionally, transduction units, substrate materials, and signal processing modules ensure accurate detection and data interpretation, while interfaces for sample handling, data output, and system encapsulation enhance usability, integration, and durability.

**Table 1:** Key components of LOC systems incorporating photonic biosensors: functions, descriptions, and representative materials.

Component	Description	Examples/Materials
Microfluidic Network [91,92]	Engineered microchannels that manipulate and transport nanoliter to microliter volumes of fluids.	Polydimethylsiloxane (PDMS), glass, PMMA; integrated with valves and pumps.
Biorecognition Element [93,94]	Molecular entity that provides specificity by selectively interacting with the target analyte.	Antibodies, aptamers, enzymes, nucleic acids (DNA/RNA), and molecularly imprinted polymers.
Transduction Unit [95,96]	Converts the biorecognition event into a quantifiable physicochemical signal.	Electrochemical (amperometric, potentiometric), optical (SPR, fluorescence), and piezoelectric.
Substrate/Platform Material [33,36,97]	Structural base that supports microfabricated components and defines the chip architecture.	Silicon wafers, glass slides, and thermoplastics (e.g., cyclic olefin copolymer-COC).
Signal Processing Module [42]	Amplifies, filters, and digitizes the transduced signal for analysis and interpretation.	Analog front ends, microcontrollers, and signal conditioning circuits.
Sample Handling Interface [98]	Facilitates introduction, distribution, and sometimes pre-treatment of biological samples.	Microreservoirs, capillary inlets, filters, micromixers.
Data Acquisition and Output Unit [68,99]	Interfaces with user or external devices for data visualization or transmission.	Integrated displays, wireless communication (Bluetooth, NFC), and smartphone integration.
Encapsulation and Packaging [100,101]	Protects sensitive components, ensures biocompatibility, and facilitates safe handling.	Biocompatible polymers, epoxy resins, hermetic seals, and micro-packaged enclosures.

**Figure 7:**
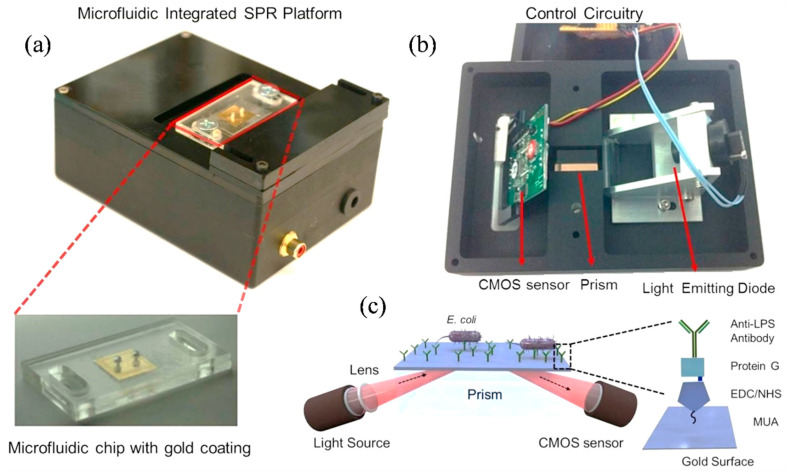
(**a**) Disposable microfluidic chips with pre-activated surfaces were mounted on the top of the device. Each chip includes inlet/outlet ports and a 50 nm gold-coated glass substrate. (**b**) The device’s optical system, shown from the bottom, consists of an LED light source, a cylindrical lens for collimation, and a rectangular prism for light guidance. Reflected light is detected by a CMOS sensor and transmitted to a portable computer via control electronics. The microfluidic chip is placed atop the prism with refractive index-matching oil to ensure optical contact. (**c**) The SPR sensing surface is functionalized with MUA, EDC, NHS, and anti-LPS antibodies for selective *E. coli* capture. Binding events alter the local refractive index, producing changes in reflected light intensity, which are recorded by the sensor and analyzed digitally [102].

Tokel et al. introduced a compact, affordable, and multiplex-capable microfluidic platform integrated with SPR sensing for the rapid detection of bacterial pathogens [102]. The custom-built SPR platform was designed for microfluidic integration (Figure 7a,b) and operated using the Kretschmann configuration, which couples light to the surface plasmon through prism excitation (Figure 7c). A collimated LED light source (λ = 705 nm) was focused by a cylindrical lens (f = 15 mm) and directed through an N-BK7 glass prism (n = 1.51) to illuminate the microchip surface. The prism, mounted on a stage, allowed for easy placement of microfluidic chips. The reflected light was captured by a CMOS sensor (500 × 582), positioned parallel to the reflected light path. The system’s optical and electrical components were housed in a portable unit measuring 13.5 × 10 × 5.2 cm, weighing 0.85 kg (Figure 7a,b). Disposable microfluidic chips were placed on the prism with an index-matching liquid (n = 1.5000 ± 0.0002) to ensure optimal light transmission (Figure 7a). A custom software package processed the sensor data, calculated real-time resonance angles, and generated resonance curves and sensograms for kinetic analysis [102].

The platform demonstrated effective capture and quantification of *Escherichia coli* (*E. coli*) in both phosphate-buffered saline (PBS) and peritoneal dialysis (PD) fluid, detecting concentrations ranging from approximately 10^5^ to 3.2 × 10^7^ CFU/mL. Furthermore, the system’s multiplexing capabilities and specificity were evaluated using *Staphylococcus aureus* (*S. aureus*) samples, confirming its ability to detect multiple bacterial targets with high accuracy. Overall, this SPR-based microfluidic device offers a promising solution for on-site pathogen detection and holds potential for broader implementation in POC diagnostics within both clinical and field settings [102].

## 4. Technological Advances

Recent technological innovations have significantly enhanced the capabilities of LOC systems, particularly through the integration of advanced photonic components. These developments are reshaping the field of biosensing by enabling higher sensitivity, real-time detection, and multiplexed analysis within compact platforms. Key progress areas include silicon photonics, which allows for scalable and cost-efficient fabrication; optofluidic photonic crystal cavities, which boost light–matter interaction for ultra-sensitive detection; and integrated detector arrays, which support simultaneous analysis of multiple targets with high spatial resolution. Furthermore, compatibility with CMOS processes has opened the door to full integration of photonic and electronic functionalities on a single chip. Together, these advances are laying the foundation for a new generation of high-performance LOC devices suitable for a wide range of applications, including clinical diagnostics, environmental monitoring, and chemical analysis [103,104,105].

### 4.1. Silicon Photonics

Recent advancements in silicon photonics have greatly improved the performance and integration of photonic biosensors within LOC systems [106,107,108]. Silicon-based materials provide several key benefits, including compatibility with existing semiconductor fabrication processes, which enables the mass production of biosensors at reduced costs [109]. This integration also allows for the seamless combination of photonic devices with electronic components, leading to highly compact and efficient LOC systems. One of the major advantages of silicon photonics is its scalability; by leveraging established semiconductor infrastructure, it is possible to produce sophisticated sensors in large volumes.

In addition to silicon, silicon nitride(Si_3_N_4_) has become an important material in silicon photonics [21,110]. Si_3_N_4_ offers distinct advantages such as low optical loss and a wide transparency range from visible to infrared wavelengths, making it ideal for use in photonic components like waveguides, resonators, and filters [111]. The ability to integrate both silicon and Si_3_N_4_ in hybrid photonic devices allows for the optimization of performance across various applications [112]. The combination of these materials leverages the strengths of each, providing both precise control over light propagation and enhanced optical properties, particularly in biosensing applications [113]. Butt et al. examined the wavelength-dependent sensitivity of a Si_3_N_4_ ridge waveguide, combining numerical analysis with experimental validation [8]. The sensitivity of a racetrack ring resonator based on the Si_3_N_4_ waveguide was experimentally assessed. The results showed that sensitivity increases from 116.3 nm/RIU to 143.3 nm/RIU as the wavelength shifts from 1520 nm to 1600 nm, demonstrating enhanced performance at longer wavelengths and highlighting the device’s potential for high-sensitivity applications [8].

Furthermore, the high refractive index contrast in silicon supports the development of small, high-performance photonic components [17,106], while Si_3_N_4_ contributes to reducing optical losses and enabling more complex, multi-material photonic circuits [114]. These capabilities make silicon photonics, especially when coupled with Si_3_N_4_, a highly promising approach for enhancing the cost-effectiveness, performance, and versatility of LOC platforms, particularly in point-of-care applications.

### 4.2. Optofluidic Photonic Crystal Cavities

Optofluidic photonic crystal cavities represent a cutting-edge fusion of photonic crystal technology and microfluidics, enabling highly sensitive and specific biomolecule detection [40,115]. These cavities feature periodic nanoscale structures that manipulate light to create resonant modes, significantly enhancing light–matter interactions [116]. When integrated into microfluidic systems, these structures amplify the interaction between target biomolecules and light, allowing for the detection of extremely low concentrations [117]. The sharp resonant peaks of photonic crystal cavities offer exceptional sensitivity and specificity, making them ideal for applications such as pathogen detection, biomarker identification, and single-molecule sensing [115].

A miniaturized vapor sensor was designed using polymer-coated, two-dimensional photonic crystal slabs that are free of structural defects [118]. The sensing approach relied on detecting shifts in the optical resonance—specifically Fano resonance—caused by molecular interactions that alter both the thickness and refractive index of the polymer layer. To evaluate the sensor’s behavior, theoretical models were used to independently analyze the contributions of refractive index variation and film thickness changes. Experiments were carried out using three different thicknesses of OV-101 polymer coatings, with sensor responses tested against hexane and ethanol vapors. Results showed that hexane generated a response approximately four times more pronounced than ethanol. Additionally, thicker polymer coatings led to increased sensitivity across both vapors, although this enhancement came at the cost of slower response times. These outcomes demonstrate the sensor’s adaptability through coating design and its potential for effective detection of organic vapors [118].

Moreover, incorporating these cavities into LOC platforms enables the development of highly sensitive biosensors capable of detecting minute biological changes. For instance, Kim et al. introduced a new class of photonic crystal nanolasers embedded within microfluidic chips, produced through multilayer soft lithography techniques [119]. This design enabled stable continuous-wave lasing at room temperature by coupling a photonic crystal nanocavity with a microfluidic flow channel. The circulating fluid enhanced thermal dissipation and simultaneously adjusted the local refractive index contrast, which was crucial for efficient laser performance. The system also allowed for real-time tuning of both the resonance wavelength and far-field emission profile by incorporating a bottom reflector. By directing fluids with varying refractive indices across this reflective layer, optical characteristics can be dynamically modified. Importantly, maintaining a separation between the cavity and reflector approximately equal to the emission wavelength enabled highly directional and efficient light output. These nanolaser systems provide a robust platform for precise biological and chemical analysis and are well-suited for integration into compact analytical devices such as LOC and micro-total-analysis systems [119].

### 4.3. Integrated Detector Arrays

Integrating detector arrays into photonic biosensors represents a significant advancement in achieving high spatial resolution and enabling multiplexed analysis [11,120,121]. By embedding photodetector arrays directly onto the chip, multiple signals can be captured and analyzed simultaneously from various regions within the microfluidic channel [122]. A low-cost, label-free photonic waveguide biosensor with multi-analyte detection was developed using a silicon photonics integrated circuit fabricated through a commercial CMOS process [123]. At the core of this system was the Local Evanescent Array Coupled (LEAC) biosensor, which operated based on a unique sensing mechanism distinct from traditional evanescent field sensors. When a biological nanofilm forms on the upper surface of the waveguide, it causes a localized change in the refractive index. This, in turn, modulated the evanescent field, which was then detected by an array of photodetectors embedded beneath the waveguide. The sensor demonstrated a photocurrent sensitivity of 20% per nanometer in response to bovine serum albumin layers thinner than 3 nm. Additional experiments using patterned photoresist, along with simulations based on the beam propagation method, further confirm the sensor’s operating principle. The design enabled full integration of optical and electronic components on a single chip, supporting compact, scalable, and multiplexed biosensing applications [123].

### 4.4. Complementary Metal-Oxide-Semiconductor (CMOS) Compatibility

CMOS compatibility plays a crucial role in integrating photonic biosensors into LOC systems [21,124,125]. The fabrication of PICs using CMOS technology involves adapting traditional semiconductor processes to integrate photonic components with electronic circuits on a single chip [126,127]. The process begins with a silicon wafer, where a layer of silicon dioxide is first grown to act as an insulating layer. A layer of silicon is then deposited on top, which will serve as the core material for the photonic waveguides. Photolithography is used to define the structure of the photonic components, such as waveguides, modulators, and detectors, by patterning the silicon layer [128]. After patterning, the silicon layer is etched to create the desired photonic structures, and additional layers of material, such as silicon nitride or aluminum oxide, may be deposited to enhance light confinement or reduce optical loss. Doping is performed to modify the refractive index of specific regions, enabling light propagation or switching functions. Metal layers are then added to form the electrical connections for integrated photonic-electronic devices. The CMOS-compatible fabrication process also includes steps such as oxidation, deposition, and etching, which are repeated to build up the photonic circuits. This process allows for high-volume production of integrated photonic circuits with low cost and compact size, enabling the development of devices for applications like optical communication, sensing, and integrated photonic systems [129].

## 5. Applications

In this section, we explore the practical applications of integrated photonic biosensors in real-world settings, demonstrating their significant potential beyond laboratory environments. By combining advanced optical components with microfluidic networks, these platforms tackle a wide range of challenges across multiple sectors. First, their role in medical diagnostics (Section 5.1) is examined, where rapid, sensitive, and portable testing has become essential. Next, environmental monitoring (Section 5.2) is reviewed, highlighting the on-site detection of pollutants and pathogens with high precision. Finally, food quality assurance (Section 5.3) applications are described, illustrating how these sensors enable fast, multiplexed screening for contaminants and allergens. Together, these examples underscore the broad versatility of LOC photonic sensors in transforming diagnostic workflows and strengthening monitoring capabilities across diverse industries.

### 5.1. Medical Diagnostics

LOC photonic biosensors are transforming medical diagnostics by combining high sensitivity, speed, and compact design into a single, efficient platform [130]. These systems use advanced photonic components like optical waveguides, resonators, and interferometers to detect subtle changes in light caused by interactions with specific biomolecules [131]. This allows for real-time, label-free detection of disease markers, pathogens, and other biological targets at extremely low concentrations [131,132]. Their miniaturized design not only reduces the need for large sample volumes and reagents but also supports portability, making them ideal for point-of-care testing. Ultimately, these biosensors hold great promise for improving early diagnosis, enabling personalized treatment, and expanding access to quality healthcare, especially in remote or resource-constrained environments (Table 2) [133].

LOC technologies have emerged as a promising solution for advancing diagnostic capabilities, offering the potential to perform complex analyses on a single, compact platform. Their appeal lies in several advantages: decentralization of laboratory testing, faster processing times, reduced costs, and the ability to run multiple assays with minimal sample volumes. The small scale of these devices allows for enhanced control over molecular interactions near the sensor surface, improving analytical precision. Technological progress in areas such as transducer development, microfluidic integration, and surface functionalization has significantly improved the performance of LOC systems. Despite these advancements, challenges persist in unifying these components into a cohesive, high-performance chip that is cost-effective, scalable, and capable of delivering reproducible results. To address these limitations, a project has been initiated to design and fabricate a silicon-based photonic biosensor with multiplexing capability. The proposed sensor featured multiple detection channels aimed at achieving high sensitivity and selectivity in antigen detection, surpassing current state-of-the-art systems [134]. As a proof of concept, the device targets three clinically relevant biomarkers: C-reactive protein (CRP), lipocalin, and tumor necrosis factor (TNF). These analytes presented varying detection challenges due to their broad concentration ranges—from milligrams per milliliter to picograms per milliliter—and the need for high specificity. The sensor utilized photonic crystal resonators, which act as wavelength-selective drop filters, enabling simultaneous detection through a single input/output configuration. Each resonator was individually optimized with specific surface chemistries, allowing tailored limits of detection and dynamic ranges for each biomarker [134].

Microphysiological systems (MPSs), also known as tissue chips (TCs) or organ-on-chip (OoC) devices, are engineered to emulate key aspects of human physiology at a microscale. These platforms aim to provide more consistent and human-relevant data compared to traditional animal models, while also offering significant ethical and economic advantages. Despite their promise, most existing TC systems rely on endpoint assays, which limit the ability to capture dynamic biological responses as they occur. To address this challenge, Cognetti et al. integrated photonic biosensors into a TC model to enable continuous, real-time monitoring of inflammatory cytokines [135]. In this study, human bronchial epithelial cells were cultured within a microfluidic chip and stimulated with lipopolysaccharide, triggering cytokine secretion that was detected in real time by the embedded sensors. Additionally, the capability to monitor analyte transport was demonstrated across the chip in response to tissue barrier disruption. This work represented the first successful application of photonic sensing technology within a human TC platform and opens new avenues for real-time analysis in drug development and disease modeling.

**Figure 8:**
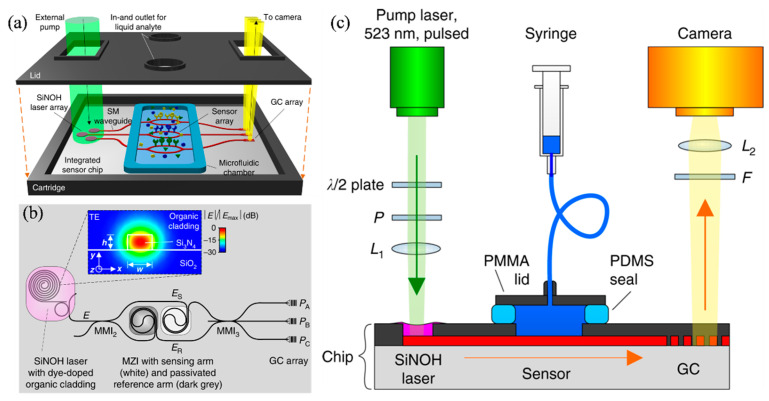
(**a**) The Si₃N₄ chip integrates a SiNOH laser array with MZI sensors, housed within a cartridge featuring optical windows for pumping and read-out. A microfluidic chamber—formed by the chip surface, cartridge lid, and a blue elastic seal—handles the analyte flow. External light pumps the SiNOH lasers using a large beam spot, eliminating the need for precise alignment. Sensor output light is directed to a read-out camera via grating couplers. (**b**) The SiNOH laser uses an open-ended spiral waveguide coupled to a ring resonator, which feeds light into the MZI sensor. Resonant coupling in the ring enables narrowband reflection from the spiral’s outer end, while broadband feedback from the inner end results from waveguide-end reflection and scattering along the 20 mm spiral. A dye-doped PMMA cladding (magenta) provides optical gain. The inset shows the waveguide cross-section with the simulated quasi-TE mode. The MZI sensor consists of a 2 × 2 MMI splitter and a 3×3 MMI combiner. Light is split between two spiral arms: the sensing arm (white) exposed to the analyte, and the reference arm (dark grey) passivated. Outputs are collected via a grating coupler array and captured by a camera. (**c**) The SiNOH laser is pumped by a pulsed laser focused with lens L_1_, with pulse energy controlled via a rotatable half-wave plate and fixed polarizer. Emitted light is coupled into an on-chip waveguide (red) and directed to the sensor. At the output, a GC array emits light upwards, filtered by a long-pass filter to block pump stray light. Lens L_2_ focuses the signal onto a CCD camera. Analyte is delivered at 0.6 mL/s through a fluidic chamber formed by the chip, a PMMA lid, and a PDMS seal [136].

Affordable and timely disease diagnosis is becoming increasingly important for personalized healthcare and public health management. Optical biosensors based on Si₃N₄ waveguide platforms offer a compelling solution by enabling highly sensitive, multiplexed detection of biomarkers using visible-wavelength light. These platforms are well-suited for scalable and cost-effective production; however, integrating low-cost on-chip light sources remains a major challenge. To address this, Kohler et al. introduced a novel biosensing approach that combined passive Si₃N₄ waveguide circuits with hybrid organic lasers, forming Si_3_N_4_–organic hybrid (SiNOH) devices (Figure 8a) [136]. These lasers are operated by optically pumping a dye-doped organic cladding material deposited directly on the waveguides, allowing for simple and scalable fabrication through techniques such as spin-coating, dispensing, or inkjet printing. Sensor signals can be read out efficiently using a basic camera system, supporting parallel detection.

Figure 8b illustrates the layout of the Si_3_N_4_ chip, where the SiNOH laser was built on a passive Si_3_N_4_ waveguide embedded within an optically pumpable, light-emitting cladding layer (see inset) [136]. A proof-of-concept experiment is conducted to validate the feasibility of sensor systems using SiNOH light sources, as shown in the setup in Figure 8c. The sensor successfully detected varying concentrations of fibrinogen in phosphate-buffered saline, achieving a length-normalized sensitivity of S/L = 0.16 rad nM⁻¹ mm⁻¹. This represented the first reported use of a co-integrated, low-cost organic light source in a fully integrated photonic biosensor. With its simplicity, versatility, and compatibility with mass production, this technology holds significant potential for applications in biophotonics and point-of-care diagnostics [136].

### 5.2. Environmental Monitoring

LOC photonic sensors are at the forefront of environmental monitoring technologies, offering compact, sensitive, and real-time detection capabilities for various pollutants [137,138,139]. These devices integrate microfluidic systems with optical components on a single chip, enabling precise manipulation of small sample volumes and facilitating rapid analysis [140]. The high sensitivity and selectivity of these sensors make them suitable for detecting trace levels of hazardous substances like heavy metals, pesticides, pathogens, and volatile organic compounds (VOCs) [33,92]. Their compact size, low power requirements, and potential for mass production allow deployment in remote or resource-limited settings, supporting decentralized and continuous environmental monitoring [141]. Integration with wireless data transmission and on-chip data processing enhances their utility, enabling real-time decision-making and contributing to early warning systems and environmental protection strategies.

Compact and scalable, on-chip optical gas sensors are increasingly used in IoT and point-of-care diagnostics. However, conventional absorption-based designs often suffer from weak gas absorbance and interference fringe noise, limiting their sensitivity. To address this, Yan et al. demonstrated a photothermal gas sensing approach using an integrated lithium niobate photonic platform [142]. A 2004 nm pump light propagated through a 91.2 mm rib waveguide, where its evanescent field is absorbed by CO₂ molecules. This absorption induced localized heating and a refractive index change in the waveguide. A 1550 nm probe co-propagated through the waveguide and undergoes phase modulation due to this photothermal effect, which was sensitively detected using heterodyne interferometry. As a proof of concept, the sensor achieved a CO₂ detection limit of 870 ppm, highlighting its potential for high-performance, on-chip gas sensing [142].

Hollow-core anti-resonant reflecting optical waveguides (ARROWs) offer promising capabilities for on-chip infrared gas sensing, though their adoption has been limited by fabrication complexity and polarization sensitivity. To address these issues, Min et al. proposed a simplified ARROW design using chalcogenide (ChG) anti-resonant layers, fabricated through thermal evaporation and bonded with epoxy resin, eliminating the need for wafer bonding [143]. Two configurations were evaluated: WG_A with four-sided and WG_B with three-sided anti-resonant cladding. The symmetric WG_A structure exhibited polarization-insensitive behavior, removing the requirement for external polarization control and enhancing sensor reliability under varying conditions. WG_A demonstrated a high external confinement factor (71%) and a detection limit of approximately 23 ppm for ethylene (C_2_H_2_) at 1.532 μm with an averaging time of 39.2 s. Its broadband sensing ability was further confirmed through successful detection of both C₂H₂ and methane (CH_4_) at distinct wavelengths (1.532 μm and 1.654 μm, respectively), emphasizing the potential of ARROWs for compact, multi-gas on-chip sensing applications [143].

**Figure 9:**
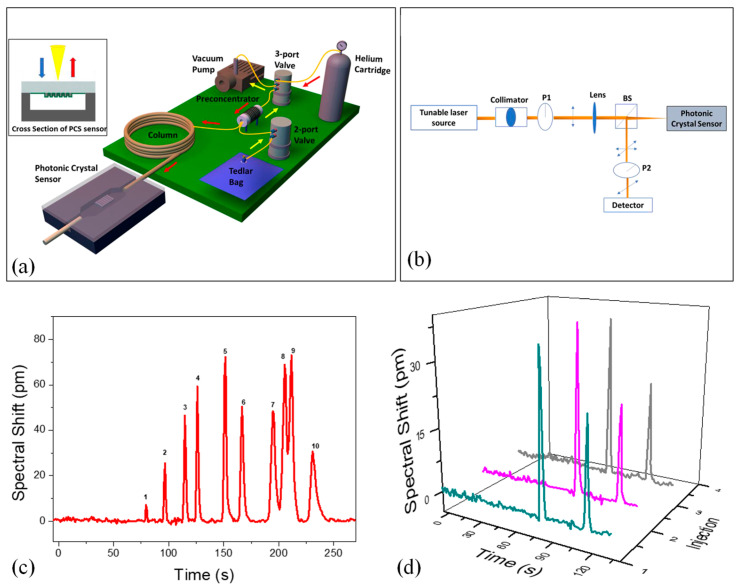
(**a**) Diagram of the integrated µGC–PCS gas analysis system. The compact µGC is housed in a 22” × 14” × 6.5” briefcase, controlled by a custom LabVIEW interface. Yellow and red arrows indicate the flow directions during sampling and analysis. The PCS sensor, transfer-printed onto glass and anodically bonded to a silicon microfluidic channel, is shown in the inset. (**b**) Optical characterization setup: BS denotes the beam splitter, and P1/P2 are linear polarizers for optical signal analysis. (**c**) Temperature-programmed chromatogram for a VOC panel with 10 VOCs on a 10 m Rtx-5MS column, using helium as the carrier gas at 1 mL/min. (**d**) Repeatability of separation shown for benzene (82 ng) and pentane (776 ng) under identical conditions over three consecutive injections [144].

Advancements in miniaturized gas chromatography (GC) have enabled rapid, on-site analysis of complex gas mixtures, making the technology more accessible for field applications. Despite progress in micro-gas chromatography (µGC), integrating a compatible and efficient detector remains a significant hurdle. To overcome this, Biswas et al. introduced a novel integration of µGC with photonic crystal slab (PCS) sensors via transfer printing [144]. This method combined the benefits of optical sensors—such as high sensitivity and fast response—with enhanced detection specificity, addressing one of the key limitations of traditional label-free optical detection. In this approach, a defect-free two-dimensional PCS was transfer-printed into borofloat glass and bonded either to a silicon-based microfluidic gas cell or directly onto a microfabricated GC column. The PCS surface was coated with a gas-responsive polymer to enable selective interactions with target analytes. Detection was achieved by monitoring spectral shifts in the Fano resonance of the PCS, allowing for real-time and quantitative analysis across a broad mass range. The µGC system, assembled with off-the-shelf components and a custom preconcentrator, was controlled via a LabVIEW program. Figure 9a shows the portable system in a 22” × 14” × 6.5” case. A mini vacuum pump draws analytes from a Tedlar bag through the preconcentrator at 5 mL/min. After sampling, helium purges unadsorbed VOCs, and the preconcentrator was heated to 330 °C in under 1.7 s, releasing analytes into the column. The preconcentrator, made from a 3.3 cm quartz capillary, uses Carbopack B and X to trap analytes. A helium flow rate of 1 mL/min was maintained. For temperature-programmed separation, a 10 m Rtx-5MS column was used, with temperature ramping from 30 °C to 90 °C. After separation, analytes were detected by a 2D PCS sensor, as shown in Figure 9b. The optical setup uses cross-polarization to detect symmetry-protected bound states in the continuum (BIC) modes.

To assess the separation performance of the µGC–PCS sensor system, a 10 VOC mixture was tested using a 10 m Rtx-5MS column under temperature-programmed conditions. Figure 9c illustrates the separation, where all peaks were symmetric with FWHM under 5.1 s. All analytes were separated within 4 min, except for ethylbenzene and p-xylene, which partially overlapped due to similar boiling points and polarities. While a lower temperature could improve their resolution, it would extend the analysis time. Alternatively, a higher flow rate or shorter column could reduce elution time, though at the cost of separation capacity. After the analytes passed through, the sensing signal returned to baseline, demonstrating complete regeneration of the PCS polymer and effective fluidics in the system. Figure 9d shows the repeatability of the µGC–PCS sensor system, tested with a mixture of benzene and pentane over three consecutive injections. Retention times, peak widths, and spectral shifts were highly consistent, with spectral shift deviations for both compounds under 4% [144].

**Figure 10:**
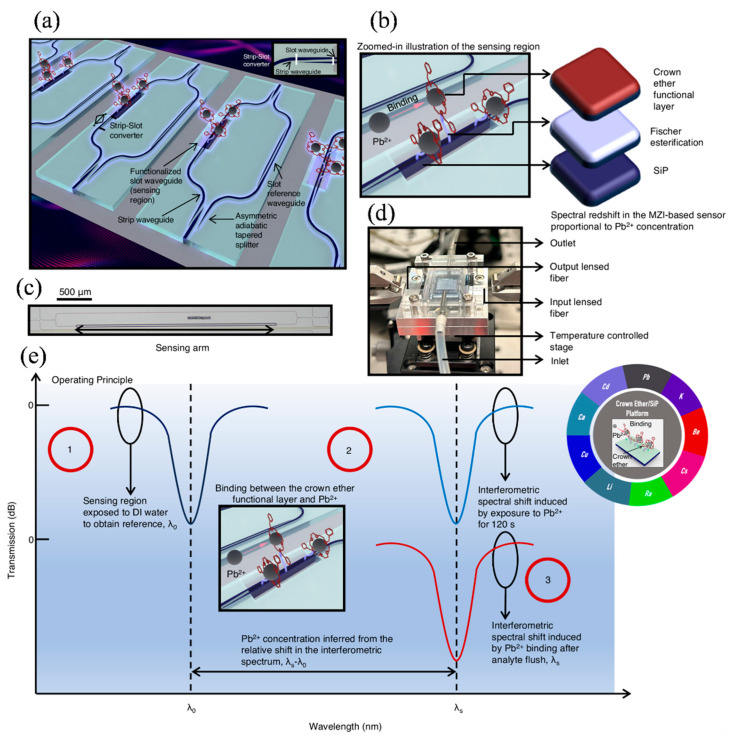
(**a**) The 3D schematic of the SiP-based Pb^2^⁺ sensor with crown ether functionalization; 20 nm SiO_2_ layer is omitted for clarity (see Figure 3a). (**b**) Zoomed-in view of the sensing region, showing Pb^2^⁺ binding to the crown ether layer. (**c**) Micrograph of the sensor with the sensing arm and 500 µm scale bar. (**d**) Assembled sensor with photonic chip and microfluidic chamber. (**e**) Operating principle of the sensor with example applications shown in the inset [146].

Water contamination detection focuses on identifying harmful substances such as bacteria, heavy metals, pesticides, or industrial chemicals in water sources. This process utilizes physical, chemical, and biological testing methods. Analytical tools like sensors, lab tests, and rapid detection techniques—such as biosensors and portable test kits—are used to measure changes in pH, turbidity, and the presence of toxins [145]. Early detection is vital to prevent health risks and ensure the safety of drinking water. Lead (Pb²⁺) contamination poses a serious, yet often overlooked, global health risk, contributing to nearly one million deaths each year. Despite its severity, effective policy measures remain lacking. In response, Ranno et al. presented an innovative silicon photonic sensing platform specifically designed for selective Pb^2^⁺ detection (Figure 10a) [146]. The system integrated crown ethers—known for their ion-selective binding capabilities—with a silicon photonic chip using an environmentally friendly Fischer esterification process (Figure 10b). The sensor’s micrograph is shown in Figure 10c. Figure 10d presents the integrated sensor setup, featuring a silicon photonic chip combined with a PDMS-based microfluidic channel, secured using a stainless-steel clamp. The channel holds up to 0.426 mL of sample solution, which was filtered through a 0.45 µm syringe filter before entering the system. Sample flow was managed via dedicated inlet and outlet tubes. Optical coupling was achieved at the chip edge using a lensed fiber (approx. 3 µm mode field diameter), aligned with a silicon taper narrowing to 175 nm. The complete setup was positioned on a thermoelectric controller (TEC), maintaining a stable temperature of 296 K with minimal thermal variation (<2 mK). Figure 10e outlines the sensor’s operating procedure. This approach not only enabled effective amine conjugation of crown ethers but also expanded the applicability of Fischer esterification beyond traditional organic systems. The resulting sensor allowed for real-time, highly selective, and quantitative ion detection, demonstrating a broad detection range and reliability in field sample testing. Moreover, the platform’s compatibility with scalable, low-cost manufacturing techniques supports its potential for widespread use in safeguarding communities from lead exposure [146].

### 5.3. Food Quality Assurance

Ensuring food safety and maintaining quality are essential to preventing foodborne illnesses [147]. Traditional testing methods rely heavily on laboratory-based analyses, which can take several days to yield results. To address the need for quicker diagnostics, techniques like PCR, ELISA, and rapid culture tests have been explored for their potential to detect pathogens more efficiently. Recent advancements have led to the development of microfluidic and LOC technologies, compact systems that enable rapid, user-friendly testing directly at the site of interest [32,148]. By integrating methods such as PCR with microfluidics, these LOC platforms offer a promising alternative or complement to conventional testing, delivering fast, accurate, and highly sensitive results on-site [50,149].

**Figure 11:**
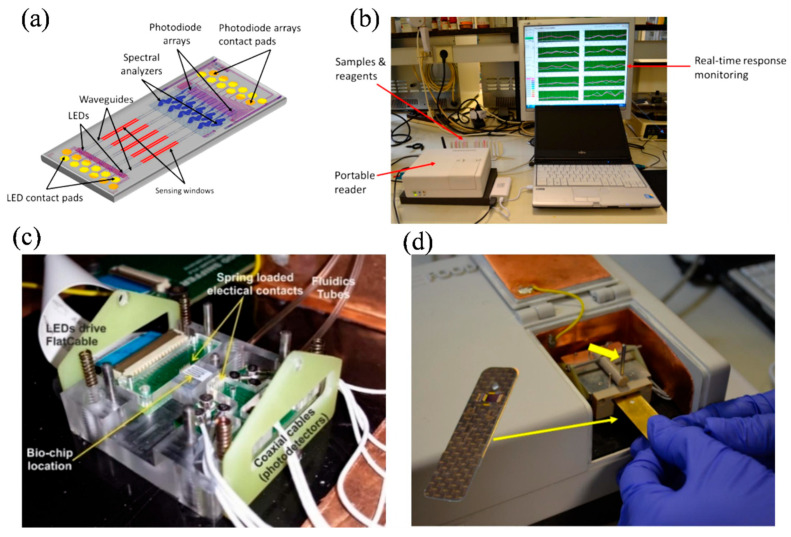
(**a**) Schematic showing the arrangement of five BB-MZI pairs, their sensing windows, LEDs, spectral analyzers, photodiode arrays, and contact pads. (**b**) Photograph of the portable reader connected to a PC for real-time response monitoring. (**c**) Photograph of the docking station’s upper part, showing the PDMS meander-like flow cell for fluidic interfacing and spring-loaded electrical contacts for LED and photodiode connections. (**d**) Image of a chip placed on the cartridge and inserted into the reader’s docking station. Fluidic and electrical connections are made by moving the lever from vertical to horizontal [152] Copyright 2024, Elsevier Ltd.

Food allergies are a global public health concern affecting people of all ages and backgrounds [150,151]. The unpredictable nature of allergic reactions complicates management, with avoidance of allergenic foods being the primary preventive measure. Food manufacturers are required to label products with allergen warnings, including phrases like “may contain traces of…” to prevent cross-contamination. While these labels help, they limit access to many processed foods for allergic individuals. A more effective solution would be to develop sensitive detection methods for allergens during production or at distribution points. Silicon chips that integrated ten MZIs, broadband light sources, spectral analyzers, and photodiode arrays were utilized for the simultaneous detection of allergens and mycotoxins [152]. As depicted in Figure 11a, the chip consists of 10 planar Si_3_N_4_ waveguides arranged in pairs, each coupled to a white light source and a photodiode array. The light source was a silicon avalanche diode, which emits light in the 530–950 nm range when reverse-biased. These waveguides are designed as BB-MZIs, with the sensing arms having a 20 × 2000 µm cladding layer opening, while the reference arms are covered. The spacing between the sensing and reference arms was 22 µm, while the distance between the sensing arms of adjacent BB-MZI pairs was 120 µm. Each pair of BB-MZIs was spaced 570 µm apart. The outputs from the 10 BB-MZIs are directed to integrated on-chip spectral analyzers, which use arrayed waveguide gratings to divide the output spectrum into 10 bands, recorded by an array of 10 photodiodes. The chip, which measures 37 mm^2^ (9.7 × 3.8 mm^2^), houses not only the optical components (LEDs, MZIs, spectral analyzers, and photodiodes) but also two sets of 12 contact pads (10 for LEDs, 10 for photodiodes, and 4 for electrical grounds). A specialized reader was designed for measurements with the integrated photonic chip (Figure 11b). It includes a docking station for electrical and fluidic connections (Figure 11c), a micropump for reagent delivery, electronics for LED power and readout, a microcontroller, and wireless communication to a PC. The chip was placed on a cartridge, inserted into the docking station, and secured with a lever (Figure 11c,d) [152].

The portable reader (20 cm × 16 cm × 7 cm) is connected to a PC running software for real-time data collection and processing. The software monitored the output spectrum shifts of the 10 BB-MZIs by recording photocurrents from the photodiodes. Phase shifts, related to analyte concentration, were calculated from the spectral changes and displayed on the interface. The output spectrum was recorded by toggling the LED every 10 s, and a discrete Fourier transform (DFT) identifies the phase shift from the main peak. This setup was employed for allergen detection in dairy rinse water and mycotoxin detection in beer through competitive immunoassays. To detect allergens, proteins such as κ-casein, peanut protein, and gliadin were immobilized on separate MZIs within a single chip. For mycotoxins, protein-conjugated mycotoxins like fumonisin B1 and deoxynivalenol were used. The process involved reacting with calibrator or sample mixtures, followed by a secondary antibody interaction to enhance the signal and reduce assay time. The allergen detection assays were completed in 10 min, with detection limits of 0.01, 0.25, and 0.05 μg/mL for κ-casein, peanut protein, and gliadin, respectively. Mycotoxin detection took 15 min, with limits of 2.0 and 10 ng/mL for fumonisin B1 and deoxynivalenol in beer. These results demonstrate the potential for efficient, sensitive, on-site multiplex detection of specific analytes [152].

**Table 2:** Comparison of sensing principles, key performance metrics, and target analytes across medical, environmental, and food applications.

Application Area	Sensing Principle	Key Metrics	Remarks
**Medical Diagnostics** [37]	Resonant (Photonic Crystal, MRR)	Sensitivity: 810 nm/RIU, 1430 pm/% NaCl Detection limit: 0.04% NaCl, 2.04 × 10⁻^5^ RIU	Multiplexed detection using cascaded microring and photonic crystal resonators
**Medical Diagnostics** [17,68]	Interferometric (MZI)	Sensitivity: 510 nm/RIU; CRP LOD: <300 pg/mL with coherent detection	Coherent detection enhances stability and sensitivity; low-cost lasers used
**Environmental Monitoring** [146]	Resonant (Si Photonic + Crown Ethers)	Broad detection range; real-time and selective detection	Ion-selective binding with crown ethers enables selective heavy metal detection
**Environmental Monitoring** [142]	Interferometric (Photothermal)	Detection limit: 870 ppm CO₂	LiNbO₃ photonic platform using heterodyne phase shift for enhanced gas sensing
**Food Safety** [152]	Interferometric (MZI Array)	Detection limits: 0.01–0.25 μg/mL (allergens) 2–10 ng/mL (mycotoxins)	Multiplexed allergen and toxin detection using integrated BB-MZI array with on-chip photodiodes

## 6. Challenges and Future Directions

Despite remarkable advancements in photonic LOC biosensors, several key challenges must be addressed to unlock their full potential in clinical, environmental, and point-of-care applications [153]. While current LOC biosensors demonstrate high sensitivity, achieving consistent detection of ultralow analyte concentrations, particularly in complex biological matrices, remains a formidable task [154,155]. Differentiating structurally similar molecules or detecting trace-level biomarkers in the presence of noise requires further enhancement of both the photonic device architecture and surface functionalization techniques. Strategies such as employing high-Q resonators, plasmonic enhancement, and engineered nanostructures (e.g., photonic crystal cavities and WGM resonators) have shown promise in improving light–matter interaction and signal-to-noise ratios. However, reproducibility and robustness across varying sample conditions still require attention, especially for point-of-care use. A recent ultra-sensitive readout method developed by Dashtabi et al. [156] offers a promising solution to reach very low detection limits while easing the fiber-chip coupling requirements.

The seamless integration of photonic components with microfluidics, electronics, and data acquisition systems continues to pose technical hurdles. Although silicon photonics and CMOS-compatible fabrication have greatly facilitated scalable integration, precise alignment, thermal management, and fluidic control within these hybrid systems remain design bottlenecks [157]. Moreover, ensuring minimal optical loss and preserving the integrity of optical signals as they traverse microfluidic environments requires further innovation in waveguide design and on-chip optics packaging. A major challenge in transitioning LOC devices from research settings to commercial applications is the absence of standardized fabrication protocols. Inconsistencies in materials, processing methods, and device architecture hinder reproducibility and batch-to-batch reliability. To enable broader adoption, particularly in regulated clinical environments, it is essential to standardize elements such as materials (e.g., Si_3_N_4_, SU-8), surface chemistry protocols, and optical calibration procedures [158,159,160,161]. While adapting commercial CMOS processes presents a promising route for scalable manufacturing, consistent design rules and clearly defined performance benchmarks are still lacking [162]. With the growing complexity and miniaturization of photonic biosensors comes the need for robust, real-time data interpretation [163]. Traditional signal processing methods often fall short when dealing with high-dimensional, noisy, and nonlinear sensor data. Therefore, there is a critical need for implementing advanced computational models, particularly those based on machine learning (ML), to extract meaningful insights from biosensor outputs. AI-driven calibration, anomaly detection, and predictive analytics will not only enhance accuracy but also enable self-correcting and autonomous biosensing systems.

**Figure 12:**
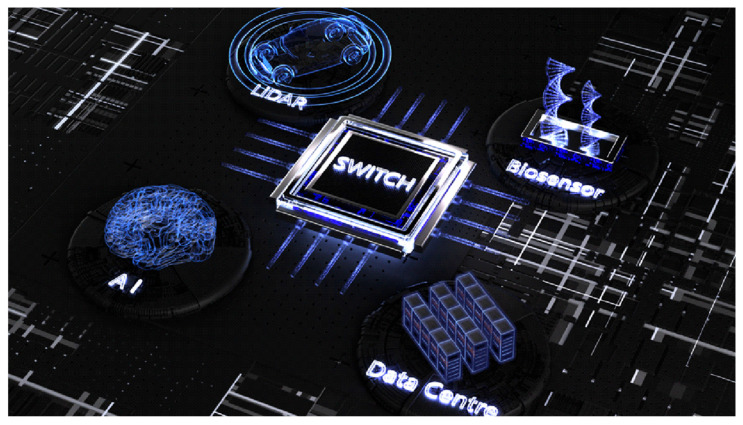
PICs with integrated on-chip laser sources enable a wide range of applications, including LiDAR systems, biosensing platforms, AI technologies, and data center communications [173].

Emerging materials such as two-dimensional semiconductors (e.g., MoS_2_, graphene), hybrid perovskites, and functionalized polymers are redefining the landscape of photonic biosensor technology [164,165]. These materials exhibit unique optical, electrical, and mechanical properties that traditional materials often lack. For instance, 2D materials offer strong light–matter interactions at the nanoscale, enabling ultra-sensitive detection through enhanced light confinement and surface plasmon resonance effects [166,167]. Hybrid perovskites provide high absorption coefficients and tunable bandgaps, which are advantageous for on-chip light sources and detectors [168,169]. Functionalized polymers, on the other hand, can be engineered for selective analyte binding, improved biocompatibility, and flexible integration with microfluidic systems [170,171,172]. Yang et al. discussed the ongoing advancements in state-of-the-art on-chip light sources with a focus on their integration into silicon-based PICs (Figure 12) [173]. The direct epitaxy of Si-based III–V quantum dot (QD) lasers has been identified as a promising approach for achieving reliable and power-efficient on-chip laser sources that offer low cost and high integration density for future Si-based PIC applications [174,175]. While notable progress has been achieved in the commercialization of these PICs for optical interconnects and sensing technologies [176], further innovations in on-chip laser technology remain essential to unlock their full potential in cutting-edge fields such as integrated quantum photonics (IQPs) and optical computing [11,12,177,178].

ML algorithms, especially deep learning and edge-based processing, are set to revolutionize LOC biosensing [179,180]. Real-time pattern recognition, adaptive filtering, and predictive diagnostics can be embedded into portable devices, offering rapid, decentralized decision-making without reliance on external computing infrastructure [181]. The integration of microfluidics with ML offers a powerful pathway for advancing high-throughput biological analysis [182]. While microfluidic systems can perform extensive experimental tasks at the microscale, their widespread adoption is often constrained by the challenges of managing and interpreting large volumes of complex data. To overcome these limitations, Mencattini et al. introduced an ML-enhanced microfluidics (MLM) platform designed to improve the diagnostic capabilities of lab-on-a-chip devices [183]. This approach leveraged deep learning methodologies to analyze cellular features in a more detailed and quantitative manner, moving beyond traditional image-based assessments of cell morphology. This study illustrates the potential of combining microfluidic diagnostics with ML to deliver reliable, scalable, and automated solutions for disease detection. By enhancing data interpretation, this hybrid approach paved the way for broader adoption of microfluidics in biomedical research and clinical diagnostics.

The convergence of photonics with soft electronics and stretchable substrates is opening new frontiers in wearable biosensing [184,185]. Photonic-based flexible sensors capable of continuous, non-invasive monitoring of physiological markers (e.g., sweat metabolites, interstitial glucose) are being developed for personalized healthcare applications [186,187,188]. Their lightweight, conformable designs make them ideal for daily use in clinical and fitness settings [189]. To address healthcare disparities, future LOC photonic biosensors must be affordable, easy to use, and operable in resource-constrained environments. Emphasis is placed on passive alignment, self-contained fluidics, and minimal user intervention. Techniques such as roll-to-roll printing [190], 3D printing, and open-source hardware platforms could democratize access to high-quality diagnostics worldwide.

Envisioning the future, several novel architectures are poised to transform integrated photonic biosensing. Hybrid plasmonic-photonic waveguides, which combine the tight field confinement of plasmons with low-loss photonic circuits, offer dramatically enhanced sensitivity at the single-molecule level [191,192,193]. On-chip quantum-dot [194] and perovskite nanolaser sources [195] promise full integration of light generation, switching, and detection on a single CMOS-compatible platform. MEMS-tunable resonators will enable real-time, adaptive spectral alignment for multiplexed assays without external tuning hardware [196,197]. Flexible and stretchable photonic circuits fabricated on polymer substrates will open the door to conformal, wearable diagnostic patches capable of continuous health monitoring [185,198]. Finally, the co-design of programmable metamaterial cavities with machine-learning algorithms will allow user-defined spectral responses and self-optimizing sensor performance [199]. Together, these design paradigms will drive the transition from laboratory prototypes to robust, scalable, and field-deployable photonic biosensing platforms.

## 7. Conclusions

Integrated photonic biosensors are driving transformative changes in lab-on-a-chip technologies by combining high sensitivity, fast response, and compact design into a unified platform. Throughout this review, we have examined the working principles behind these devices, including evanescent field sensing, resonant photonic structures, and interferometric techniques, which enable label-free, real-time detection of biomolecules with high precision. By integrating these photonic elements with advanced microfluidic systems, researchers have created highly miniaturized diagnostic platforms capable of performing complex biochemical assays using minimal sample volumes. Materials such as silicon and silicon nitride, alongside CMOS-compatible fabrication processes, have played a pivotal role in enabling scalable and cost-effective manufacturing.

What distinguishes the current evolution of this field as “next-generation” is the emergence of technologies that significantly expand the capabilities of integrated biosensors. These include on-chip photonic crystal nanolasers, optofluidic photonic crystal cavities, AI-assisted signal processing, and fully integrated detector arrays for parallel analysis. Together, these advancements push beyond traditional limitations by enabling higher sensitivity, enhanced multiplexing, and real-time performance in formats suitable for decentralized and point-of-care use.

While challenges remain, such as handling complex biological samples, achieving uniformity in fabrication, and optimizing sensor robustness. The convergence of photonics with AI, flexible electronics, and novel materials like two-dimensional semiconductors is creating a powerful foundation for the next wave of diagnostic technologies. In essence, integrated photonic biosensors are no longer just miniaturized analytical tools; they are evolving into intelligent, autonomous, and versatile systems that promise to redefine diagnostics in healthcare, environmental monitoring, and food safety. These developments underscore the article’s focus on next-generation platforms that are poised to deliver accessible, high-performance solutions for real-world applications.
